# A Series of Personalized Virtual Light Therapy Interventions for Fatigue: Feasibility Randomized Crossover Trial for N-of-1 Treatment

**DOI:** 10.2196/45510

**Published:** 2023-09-18

**Authors:** Mark Butler, Stefani D’Angelo, Heejoon Ahn, Thevaa Chandereng, Danielle Miller, Alexandra Perrin, Anne-Marie N Romain, Ava Scatoni, Ciaran P Friel, Ying-Kuen Cheung, Karina W Davidson

**Affiliations:** 1 Institute of Health System Science Feinstein Institutes for Medical Research Northwell Health New York, NY United States; 2 Gordon F. Derner School of Psychology Adelphi University Garden City, NY United States; 3 Mailman School of Public Health Columbia University New York, NY United States; 4 Donald and Barbara Zucker School of Medicine at Hofstra/Northwell Northwell Health Hempstead, NY United States

**Keywords:** virtual light therapy interventions, fatigue, light therapy, primary care, feasibility, acceptability, effectiveness, usability, seasonal affective disorder, phototherapy, photoradiation, photochemotherapy, color therapy, heliotherapy, photothermal therapy, UV therapy, chromotherapy, color light therapy, mobile phone

## Abstract

**Background:**

Fatigue is one of the most common symptoms treated in primary care and can lead to deficits in mental health and functioning. Light therapy can be an effective treatment for symptoms of fatigue; however, the feasibility, scalability, and individual-level heterogeneity of light therapy for fatigue are unknown.

**Objective:**

This study aimed to evaluate the feasibility, acceptability, and effectiveness of a series of personalized (N-of-1) interventions for the virtual delivery of bright light (BL) therapy and dim light (DL) therapy versus usual care (UC) treatment for fatigue in 60 participants.

**Methods:**

Participants completed satisfaction surveys comprising the System Usability Scale (SUS) and items assessing satisfaction with the components of the personalized trial. Symptoms of fatigue were measured using the Patient-Reported Outcomes Measurement Information System (PROMIS) daily, PROMIS weekly, and ecological momentary assessment (EMA) questionnaires delivered 3 times daily. Comparisons of fatigue between the BL, DL, and UC treatment periods were conducted using generalized linear mixed model analyses between participants and generalized least squares analyses within individual participants.

**Results:**

Participants rated the usability of the personalized trial as acceptable (average SUS score=78.9, SD 15.6), and 92% (49/53) of those who completed satisfaction surveys stated that they would recommend the trial to others. The levels of fatigue symptoms measured using the PROMIS daily fatigue measure were lower or improved in the BL (B=−1.63, 95% CI −2.63 to −0.63) and DL (B=−1.44, 95% CI −2.50 to −0.38) periods relative to UC. The treatment effects of BL and DL on the PROMIS daily measure varied among participants. Similar findings were demonstrated for the PROMIS weekly and EMA measures of fatigue symptoms.

**Conclusions:**

The participant scores on the SUS and satisfaction surveys suggest that personalized N-of-1 trials of light therapy for fatigue symptoms are both feasible and acceptable. Both interventions produced significant (*P*<.05) reductions in participant-reported PROMIS and EMA fatigue symptoms relative to UC. However, the heterogeneity of these treatment effects across participants indicated that the effect of light therapy was not uniform. This heterogeneity along with high ratings of usability and satisfaction support the use of personalized N-of-1 research designs in evaluating the effect of light therapy on fatigue for each patient. Furthermore, the results of this trial provide additional support for the use of a series of personalized N-of-1 research trials.

**Trial Registration:**

ClinicalTrials.gov NCT04707846; https://clinicaltrials.gov/ct2/show/NCT04707846

## Introduction

### Background

One of the most commonly recorded patient symptoms in conversations with primary care providers is fatigue [1]; 25% of patients endorse fatigue as a complaint, and 6.5% of patients name symptoms of fatigue as their primary reason for seeking treatment [2]. Fatigue symptoms can lead to deficits in psychomotor functioning (eg, attention and vigilance), cognition (eg, memory and reasoning), work performance, and mood; an increased likelihood of workplace accidents and highway mortality; and reduced quality of life [3-9]. Fatigue symptoms are common and can have negative effects on mental health and functioning, despite the lack of established guidelines for fatigue interventions.

Fatigue may stem from many causes, one of which is the disruption of circadian rhythms that control the sleep-wakefulness cycle [10-12]. Reviews indicate that bright light (BL) therapy can reduce fatigue via two circadian rhythm mechanisms: (1) light inﬂuences the suprachiasmatic nucleus, a region in the hypothalamus that controls circadian rhythms, and (2) light has alerting effects, which in turn facilitate thalamic and cortical connections [13,14]. Light therapy interventions have demonstrated that exposure to BL can lead to reduced sleep problems and increased levels of wakefulness and alertness during the day [15-17]. Despite these promising results, light therapy interventions for fatigue have had small to moderate effects and large heterogeneity of treatment effects between participants [18-20]. This suggests that light therapy may be an efficacious intervention for fatigue symptoms but may not be equally effective for all participants.

To determine which participants may benefit from interventions with heterogeneous treatment effects such as light therapy, personalized (N-of-1) trial designs are ideal. Personalized trials have a patient-centered approach and single-case experimental research design that provides essential clinical information for selecting the best treatments for individual patients [21]. Personalized trials are designed to assist patients in making treatment decisions that are informed by high-integrity, evidence-based information examining how particular interventions influence the outcomes of interest [22]. Personalized trials have been demonstrated to help identify treatments that are effective among individual patients dealing with fatigue associated with cancer diagnoses [23-25]. Furthermore, evidence suggests that personalized trials are useful in examining the effects of light therapy on depression [26]. Individual patients can use the information from a personalized trial to identify the most effective treatment for their symptoms of fatigue. This allows patients to receive the treatment that best fits them, thereby improving outcomes and reducing overall costs resulting from the use of less optimal treatments [27].

### Objectives

This study evaluated the feasibility, acceptability, and effectiveness of a series of personalized interventions for virtual delivery of BL therapy, dim light (DL) therapy, and usual care (UC) treatment for fatigue symptoms among 60 participants. By using new wearable technologies (such as Fitbit devices [Fitbit Inc]) and commercially available light therapy devices (such as the AYO wearable device), this study allowed for continuous data collection and virtually conducted assessment [28]. Furthermore, virtual delivery of the intervention allowed each participant to receive treatment and be assessed for fatigue in their own home. The results of this study will determine whether virtual delivery of these interventions is feasible and acceptable for participants with fatigue and will allow clinicians to identify patients who may benefit most from virtual delivery of light therapy to treat symptoms of fatigue.

## Methods

### Study Design

**Figure 1:**
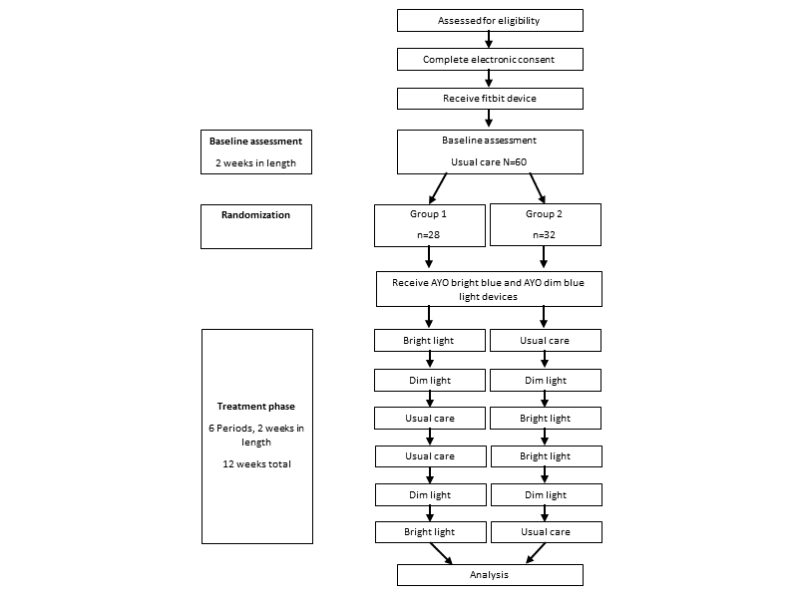
Participant timeline.

This study included 60 randomized N-of-1 trials with alternating periods of BL therapy, DL therapy, and UC. The participants took part in the trial virtually over a period of 14 weeks (Figure 1). For BL and DL therapy, the participants used 2 AYO light therapy devices. Participants were also provided with a Fitbit Charge 3 device to measure their levels of activity. Additional details regarding the study design can be found in the study protocol, which is published elsewhere [28].

The first 2 weeks of the study were a baseline assessment period. During the baseline assessment, each study participant was asked to manage fatigue symptoms as they usually do and to wear their Fitbit device at all times, including during sleep. The participants were asked to rate an ecological momentary assessment (EMA) of their fatigue symptoms, pain, concentration, stress, mood, and confidence 3 times daily via SMS text messages. Each evening, the participants answered a survey questionnaire assessing their symptoms of fatigue from that day. Each weekend, participants completed a longer survey, asking them to rate their fatigue symptoms over the course of the previous week. After successfully completing the baseline period, the participants were randomized into 2 arms with different orders of 2-week treatment blocks of BL AYO therapy, DL AYO therapy, or UC, which were each presented twice (Figure 1). At the completion of the study, each participant was provided with a satisfaction survey and a report containing their analyzed data. Study recruitment began in December 2020, and the study was successfully concluded in January 2022. The CONSORT (Consolidated Standards of Reporting Trials) and CONSORT extension for reporting N-of-1 Trials (CENT) reporting guidelines were used in this study [29].

### Study Population

All participants in the study had a minimum threshold of fatigue, defined as a score of ≥12 on the Patient-Reported Outcomes Measurement Information System (PROMIS) Fatigue Short Form 8a scale. After consultation with an ophthalmologist, the study excluded participants with a family history of Stargardt disease and those with diabetes for eye vision safety reasons. Participants were required to have a smartphone capable of receiving text messages, live in the United States, and be able to participate with blue light therapy without any medical complications. The inclusion and exclusion criteria are provided in Textbox 1.

**Inclusion and exclusion criteria.**

**Inclusion criteria**

- Are aged 18-59 y
- Are fluent in English
- Have self-reported fatigue scores of ≥12 on a modified Patient-Reported Outcomes Measurement Information System Fatigue Short Form 8a scale
- Are able to participate in blue light therapy
- Possess a smartphone capable of receiving SMS text messages
- Possess an email account that can be accessed regularly
- Live in the United States

**Exclusion criteria**

- Are aged <18 y or ≥60 y
- Are pregnant
- Have had previous diagnosis of eye disease, such as cataracts, glaucoma, macular degeneration, Stargardt or family history of Stargardt, retinitis or retinopathy, or other retinal disorders
- Have had previous diagnosis of diabetes
- Have had previous eye surgery
- Have sensitivity to light or use of medication causing sensitivity to light
- Have epilepsy or a history of seizures
- Participate in shift work (evening or night shifts, early morning shifts, rotating shifts, etc)
- Have had a previous diagnosis of a serious mental health condition or psychiatric disorder that could be exacerbated by exposure to light therapy or that would compromise their ability to engage with full consent in this trial or adhere to the protocol

### Recruitment

Participant recruitment was conducted using multiple formats (including videos, images, and text posts) and on several platforms (including Facebook, Instagram, Google, and Reddit). The content of advertisements was varied to target different subpopulations (namely, by gender or US state of residence). The recruitment of Northwell Health employees and individuals who previously expressed interest in personalized trials and the Northwell Health Clinical Trials Listing was conducted via email. Interested participants were provided with web-based information containing details about the pilot study and were asked to complete a screening questionnaire containing the study inclusion and exclusion criteria. If the participant was deemed eligible, the study staff sent them an email containing the electronic consent form and additional information within 2 business days.

### Ethical Considerations

This trial protocol and all amendments to the protocol (#20-0835) were reviewed by the Northwell Health Institutional Review Board.

Individuals eligible to participate after the screening received a message from the study staff with a link to the electronic consent form and a short video explaining the key details of the study protocol and consent form. To ensure an understanding of the protocol and consent process, the participants completed a 4-question screening measure before enrollment. Consent was obtained electronically, and a copy of the consent was electronically mailed to the participant along with the study instructions and devices (ie, Fitbit and AYO). Signed consent forms were stored electronically on a Health Insurance Portability and Accountability Act–compliant, Northwell Health–approved shared drive accessible only to the institutional review board–approved study staff. An example of a consent form can be found in Multimedia Appendix 1.

Participants were mailed a US $100 payment card after completing all study components.

In addition, participants were allowed to keep their Fitbit Charge 3 (a value of US $120) and an AYO light therapy device (a value of US $299).

To minimize loss of confidentiality, all participant information was stored in a password-protected, Northwell Health–approved, Health Insurance Portability and Accountability Act–compliant database. Only the study staff approved by the Northwell Health Institutional Review Board received access to this data. Participants were made aware of all data being collected and all technology used for data collection.

### Assignment of Interventions

Approximately half (28/60, 47%) of the sample was randomized by the study statistician to receive the protocol in the following order of balanced 2-week treatment periods: BL, DL, UC, UC, DL, and BL. The remaining 32 (53%) participants were randomized in the following order of 2-week treatment periods: UC, DL, BL, BL, DL, and UC. In both treatment arms, the participants alternated between the BL, DL, and UC periods. Randomization to the 2 treatment orders was conducted in 6 blocks using a readily accessible randomization website [30]. This randomization to the treatment order can be viewed in the participant timeline in Figure 1.

### Interventions

Participants received 2 pairs of AYO light therapy glasses, labeled “Bright” and “Dim.” AYO glasses are commercially available wearable light therapy device that use blue (mean 470, SD 2 nm wavelength) light of SD 100 Lux and irradiance of SD 250 μW/cm² [31,32]. The “Bright” light glasses were hardcoded to emit the BL therapy treatment (ie, blue light with the wavelength, light intensity, and irradiance listed in the previous sentence above). The “Dim” light glasses were hardcoded to emit the DL therapy treatment (ie, 1% regular intensity; <2 Lux). All other parameters (including wavelength and irradiance) were identical for the 2 types of glasses. The participants were not blinded to the treatment condition during their 2-week treatment periods. Participants also received a treatment schedule indicating when to use BL glasses, when to use DL glasses, and when to avoid light therapy treatments. Participants were instructed to download a unique research study application that initiated light therapy sessions at a predetermined session length of 30 minutes. During the UC period, participants were asked to refrain from engaging in any other treatment for fatigue. Additional details regarding the light therapy intervention can be found in the study protocol, which is published elsewhere [28].

### Outcomes

#### Primary Outcome

The primary outcome for this study was the average participant rating of the feasibility of the trial, measured using the System Usability Scale (SUS) [33]. The SUS is a 10-item questionnaire asking participants to rate aspects of the study on a Likert scale from “Strongly disagree” (0) to “Strongly agree” (4). Scores for each item were multiplied by 2.5 and summed to create a total score ranging from 0 to 100, with higher scores indicating a greater level of usability. The SUS has been used and validated for use in multiple programs and trials [34,35]. The SUS was used to measure the feasibility of this trial, as it is a well-validated measure that examines the usability and learnability of our personalized trial system [36-38]. Prior research has identified the goal of feasibility trials as helping to design a larger confirmatory trial [39] or identifying the possibility of conducting a particular program within certain parameters [40]. In this case, the usability and learnability of our personalized trial system for the treatment of fatigue are essential for large-scale development and validation of personalized N-of-1 trials for fatigue. Therefore, we believe that the SUS represents a widely used, validated measure that examines 2 facets of our personalized N-of-1 trials, which are key to feasibility: the usability and learnability of the system [36,41].

#### Secondary Outcomes

Secondary outcomes in this study included self-reported daily fatigue, self-reported weekly fatigue, EMA self-reported fatigue ratings, and participant ratings of satisfaction with the trial. In this study, the effects of BL and DL on daily fatigue were used to determine the effectiveness of each intervention relative to UC.

The PROMIS fatigue scales were used to measure daily levels of fatigue symptoms (PROMIS Item Bank v1.0 Fatigue 7b Daily) and weekly levels of fatigue symptoms over the past 7 days (PROMIS Item Bank v1.0 Fatigue 8a). All items were rated on a scale of 1 to 5, with higher scores indicating greater symptoms of fatigue. The PROMIS measures were collected every evening (for daily symptoms of fatigue) and on the weekends (for weekly symptoms of fatigue). For both PROMIS scales, scores were converted to *T* scores using methods from the PROMIS scoring manual based on item response theory. This allows scores to be compared with previously established population norms. With a SD of 10, a *T* score of 50 is the average for the US general population [42]. A higher *T* score indicates a higher level of fatigue. The reliability and validity of the PROMIS fatigue scales have been well supported [43].

Symptoms of fatigue were also assessed via EMA using a measure adapted from the Numeric Pain Rating Scale [44]. These assessment measures are single-item assessments administered 3 times daily via text messages asking participants to rate their fatigue in the current moment on a scale of 0 to 10. The timing of the text messages was randomized between participants’ self-reported wake and sleep times. The text message stated, “I feel fatigued,” and it asked participants to provide ratings of 0 to indicate no feeling of fatigue and scores of 1 to 3, 4 to 6, 7 to 9, and 10 to indicate a little, some, significant, and extreme feeling of fatigue, respectively. Similar to the PROMIS fatigue scales, changes in the EMA measures were examined to determine the effectiveness of each intervention.

Measures of participant satisfaction were used to determine the acceptability of the trial. Patient satisfaction with the trial was assessed using a satisfaction survey administered upon the completion of the treatment. The survey assessed participant satisfaction with elements of the trial, including the onboarding process, the consenting process, the AYO device, the Fitbit device, the N-of-1 trial design, assessment measures, and the participant report. Participant satisfaction with the interventions (both BL and DL therapies) was also assessed. Participants were asked to rate their satisfaction on a scale of 1 (“not very satisfied”) to 5 (“very satisfied”).

### Statistical Analysis

#### Sample Size Calculation

The sample size of 60 participants was chosen to measure the feasibility of this series of N-of-1 trials using the SUS. Expecting a trial completion rate of 70% with 60 randomized participants, we anticipated that SUS data would be available for about 42 participants, thus giving the study an SE of no more than 8% for a 1-sample binomial test with a 1-sided α level of 2.5% in estimating the rate of SUS≥85, an exceptional level of usability [45]. Additional data regarding sample size calculation can be found in the previously published study protocol [28].

#### Primary Analysis

The primary analysis examined participant scores on the SUS to determine the feasibility of this series of N-of-1 interventions for symptoms of fatigue. Ratings from all enrolled participants (N=60) on the SUS were summarized using descriptive statistics, including mean, SD, and distribution for the overall score as well as scores for individual items. The distribution of the SUS scores was visualized using a histogram. The SUS data were interpreted by comparing the scores for our series of N-of-1 trials with established usability standards in the SUS literature. Scores on the SUS ≥70 were used to define the trial as meeting an acceptable rating [45]. The frequencies of responses to individual SUS items were also depicted using bar charts.

#### Secondary Analyses

To determine the effectiveness of our intervention, we examined multiple self-reported measures of fatigue symptoms. We reported the means and SDs for (1) PROMIS daily fatigue scores and PROMIS weekly fatigue scores and (2) self-reported EMA fatigue scores for the baseline assessment period and each treatment period (BL, DL, and UC). We then compared the overall means of secondary outcomes for the BL, DL, and UC periods with baseline means using 2-tailed, paired sample *t* tests. Finally, the effects of each treatment on daily fatigue, weekly fatigue, and self-reported EMA fatigue were assessed using generalized linear mixed models with an autoregressive model of order 1 (AR {1}), which accounts for linear trends between fatigue ratings across time. For these analyses, we considered “week” as a linear term and a factor in the mixed model to explore the nonlinear time effects of each treatment. In addition, to determine whether BL therapy or DL therapy was superior to UC and the other light therapy for reducing fatigue among individual patients, treatment effects were assessed using an autoregressive, generalized least squares model of order 1 (AR {1}). This model includes the type of light therapy as the main exposure, adjusted for time (eg, days since enrollment) linearly as a covariate and accounted for in terms of autocorrelations of the order 1.

To determine the acceptability of the trial, we examined the means and SDs for the items on the patient satisfaction questionnaire administered at the end of the trial. Higher average scores were interpreted as higher levels of satisfaction with the trial overall and specific trial elements. Participant responses were also depicted using bar graphs to show the frequency of the responses.

## Results

### Enrollment and Sample Characteristics

**Figure 2:**
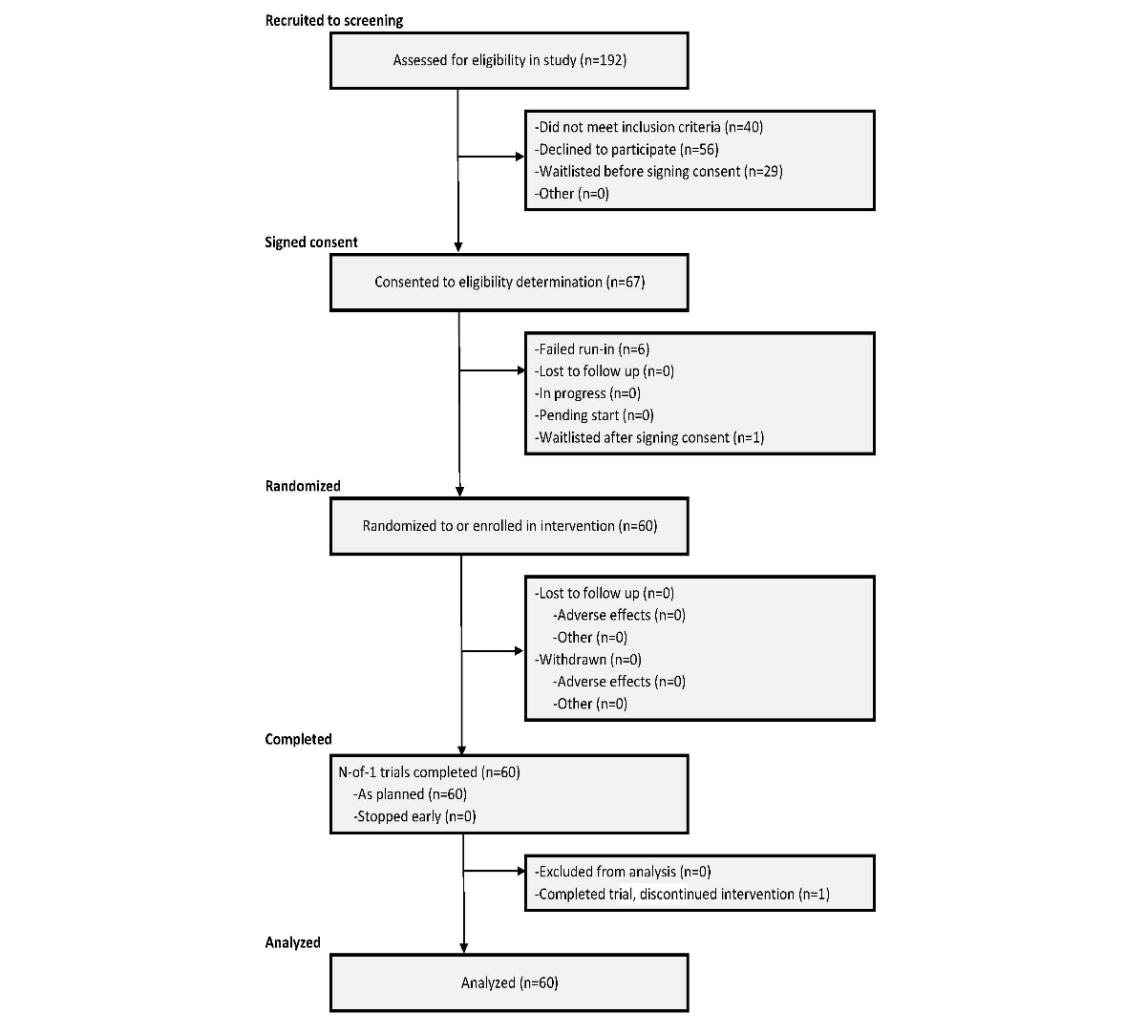
Participant CONSORT (CENT) Flow Diagram.

A total of 192 participants were screened for the study, yielding an enrolled sample of 60 participants. Additional details regarding enrollment can be found in the CONSORT diagram for this study (Figure 2). The most frequently endorsed recruitment sources were social media (42/192, 21.9%), recruitment emails within the Northwell Health system (39/192, 20.3%), and word of mouth (32/192, 16.7%). The 60 enrolled participants had a mean age of 36.6 (SD 11) years; 65% (39/60) of the participants were female, 18% (11/60) were Hispanic, and 67% (40/60) were White. Participant characteristics did not differ between the 2 treatment orders. The full characteristics of the samples are provided in Table 1.

**Table 1:** Descriptive characteristics of the sample (N=60).

Variable		Total sample	Treatment order 1 (n=28)^a^	Treatment order 2 (n=32)^b^	*P* value^c^	
Age (years), mean (SD)		36.6 (11)	35.5 (10.8)	37.7 (11.3)	.45	
**Gender, n (%)**						>.99
	Female	39 (65)	19 (68)	20 (63)		
	Male	20 (33)	9 (32)	11 (34)		
	Other	1 (2)	0 (0)	1 (3)		
**Race, n (%)**						.82
	Asian	8 (13)	5 (18)	3 (9)		
	Black	4 (7)	2 (7)	2 (6)		
	White	40 (67)	17 (61)	23 (72)		
	Mixed	5 (8)	3 (11)	2 (6)		
	Other	3 (5)	1 (4)	2 (6)		
**Ethnicity, n (%)**						.86
	Hispanic	11 (18)	5 (18)	6 (19)		
	Non-Hispanic	48 (80)	22 (79)	26 (81)		
	Unknown	1 (2)	1 (4)	0 (0)		

^a^Treatment order 1: bright light, dim light, usual care, usual care, dim light, and bright light.

^b^Treatment order 2: usual care, dim light, bright light, bright light, dim light, and usual care.

^c^*P* values for comparisons of participant characteristics between treatment orders were obtained from independent samples *t* tests for continuous variables and Fischer exact tests for categorical variables.

### SUS Overview

**Figure 3:**
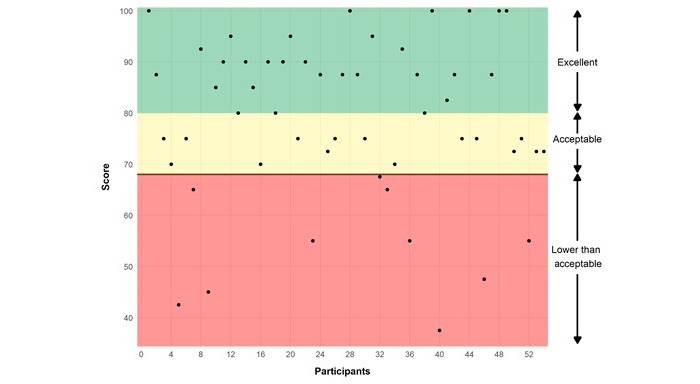
Scores on the System Usability Scale (SUS) by participants.

A total of 53 participants completed the primary outcome of the SUS. The average SUS score (78.9, SD 15.6) indicated that the trial was an acceptable system according to the SUS scoring thresholds (SUS total score ≥70) [45], and most participants (43/53, 81%) rated the trial as acceptable or better. This suggests that most individuals found the personalized N-of-1 intervention to be usable and learnable. In total, 11% (6/53) of the participants responded with the highest possible usability score. In total, 19% (10/53) of the participants rated the trial as having lower-than-acceptable levels of usability (Figure 3). The SUS scores for each participant are shown in Figure 3.

**Figure 4:**
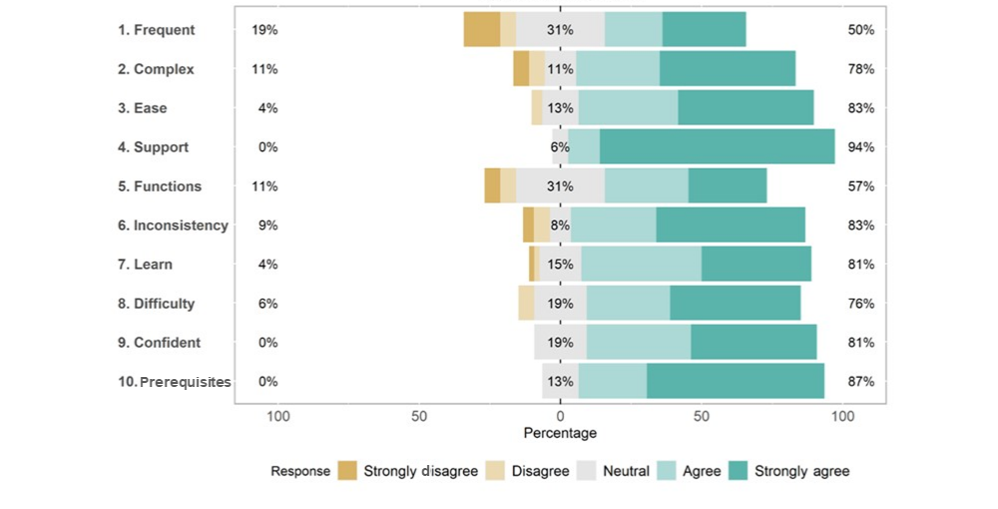
Distribution of responses for individual items on the System Usability Scale (SUS).

Average scores for individual items on the SUS (Table 2) ranged from 2.48 (SD 1.33) for the item “I think that I would like to the use this system frequently” to 3.78 (SD 0.54) for the item “I do not think that I would need the support of a technical person to be able to use this system.” Figure 4 shows the distribution of scores for each item on the SUS. All items were rated favorably by the majority of participants, with the exception of the item “I think that I would like to the use this system frequently,” which was rated favorably by 51% (27/53) of the sample. For this item, 30% (16/53) of the sample rated the statement as neutral and 19% (10/53) stated that they disagreed. In total, 81% (43/53) of the participants agreed with the statement “I felt very confident in using the system,” 19% (10/53) rated it as neutral, and none disagreed with it.

**Table 2:** Descriptive statistics for the System Usability Scale (N=60).

Measure		Values, n (%)	Values, mean (SD; range)
System Usability Scale overall score		53 (88)	78.89 (15.64; 37.5-100)
**System Usability Scale individual items^a^**			
	1. I think that I would like to use this system frequently.	53 (88)	2.48 (1.33; 0-4)
	2. I did not find the system unnecessarily complex.^b^	53 (88)	3.09 (1.15; 0-4)
	3. I thought the system was easy to use.	53 (88)	3.28 (0.83; 1-4)
	4. I do not think that I would need the support of a technical person to be able to use this system^b^	53 (88)	3.78 (0.54; 2-4)
	5. I found the various functions in this system were well integrated.	53 (88)	2.69 (1.11; 0-4)
	6. I did not think there was too much inconsistency in this system.	53 (88)	3.23 (1.07; 0-4)
	7. I would imagine that most people would learn to use this system very quickly.	53 (88)	3.15 (0.88; 0-4)
	8. I did not find the system very awkward to use.^b^	53 (88)	3.17 (0.93; 1-4)
	9. I felt very confident using the system.	53 (88)	3.26 (0.76; 2-4)
	10. I did not need to learn a lot of things before I could get going with this system.^b^	53 (88)	3.50 (0.72; 2-4)

^a^Questions were rated on a 5-point Likert scale from 0 “Strongly disagree” to 4 “Strongly agree.”

^b^Items were initially reverse coded but have been recoded to be on the same scale as other items. The text of these questions has been revised from the original items to reduce confusion.

### Participant Ratings of Fatigue

At baseline, participants endorsed moderate levels of fatigue symptoms on the PROMIS daily measure (mean 58, SD 8), PROMIS weekly measure (mean 61, SD 6), and EMA measure (mean 5.20, SD 1.74). During the intervention period, participants reported lower levels of mean fatigue symptoms during the BL, DL, and UC periods relative to baseline (Table 3).

Of the 60 participants, 56 (93%), 50 (83%), and 55 (92%) had sufficient data to evaluate the relative effectiveness of the BL and DL interventions relative to UC for daily fatigue, weekly fatigue, and EMA fatigue, respectively. Generalized linear mixed model regressions were used to determine the difference in fatigue symptoms between the BL, DL, and UC intervention periods. Symptom reductions were found in the PROMIS daily fatigue measure during BL periods relative to UC periods (B=−1.63, 95% CI −2.63 to −0.63; Table 4) and DL periods relative to UC periods (B=−1.44, 95% CI −2.50 to −0.38). Symptoms of fatigue did not differ on the PROMIS daily fatigue measures between the DL and BL periods (B=0.20, 95% CI −0.63 to 1.03). Similar findings were present in the PROMIS weekly fatigue measure for BL versus UC periods (B=−2.48, 95% CI −3.78 to −1.19), DL versus UC periods (B=−2.25, 95% CI −3.32 to −1.18), and DL versus BL periods (B=0.15, 95% CI −0.84 to 1.14). Finally, the same pattern of results was found for the EMA fatigue symptoms for BL versus UC periods (B=−0.31, 95% CI −0.57 to −0.05), DL versus UC periods (B=−0.31, 95% CI −0.52 to −0.11), and DL versus BL periods (B=−0.01, 95% CI −0.17 to 0.16).

**Figure 5:**
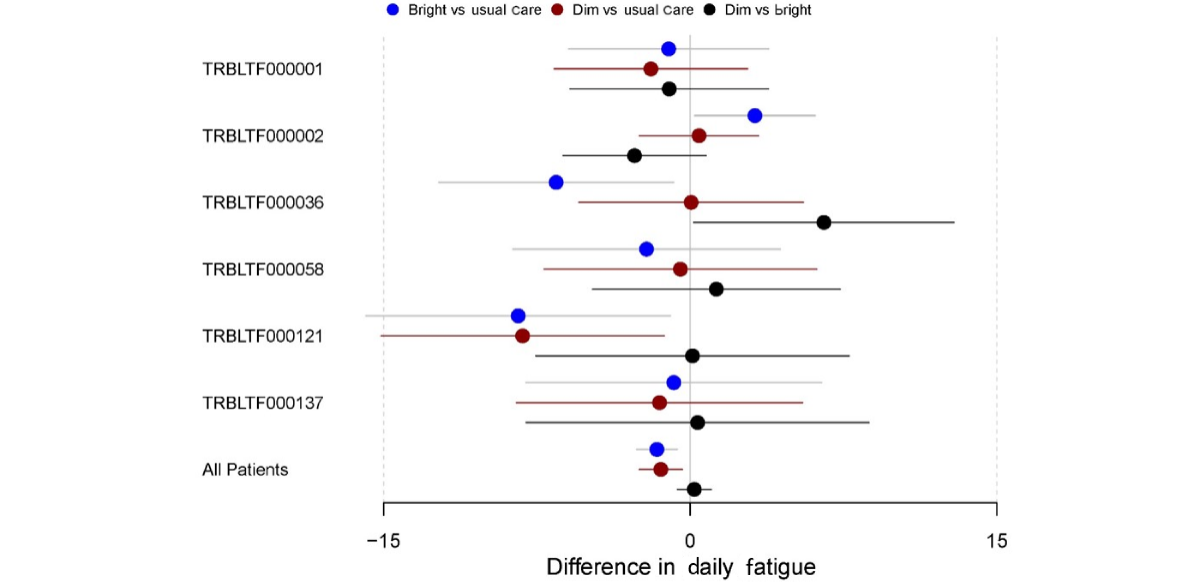
Difference in the Patient-Reported Outcomes Measurement Information System (PROMIS) daily fatigue by intervention period (subsample of participants). Participant IDs are denoted with their deidentified study code (eg, TRBLTF000001).

**Figure 6:**
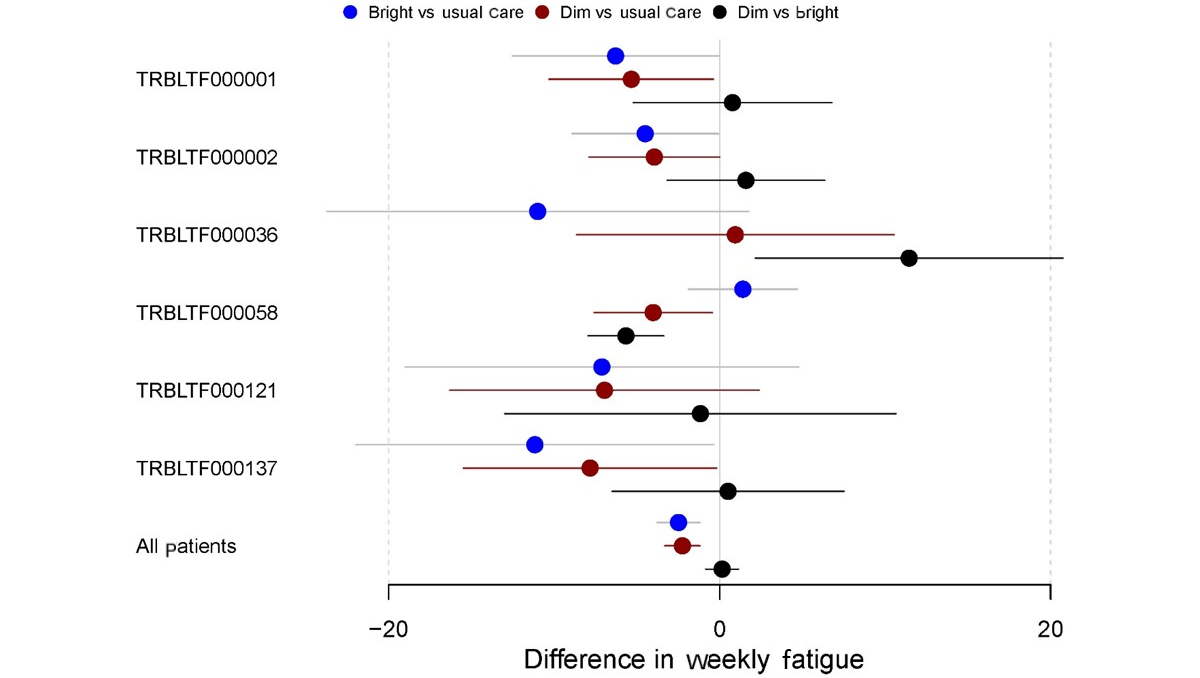
Difference in the Patient-Reported Outcomes Measurement Information System (PROMIS) weekly fatigue by intervention period (subsample of participants). Participant IDs are denoted with their deidentified study code (eg, TRBLTF000001).

**Figure 7:**
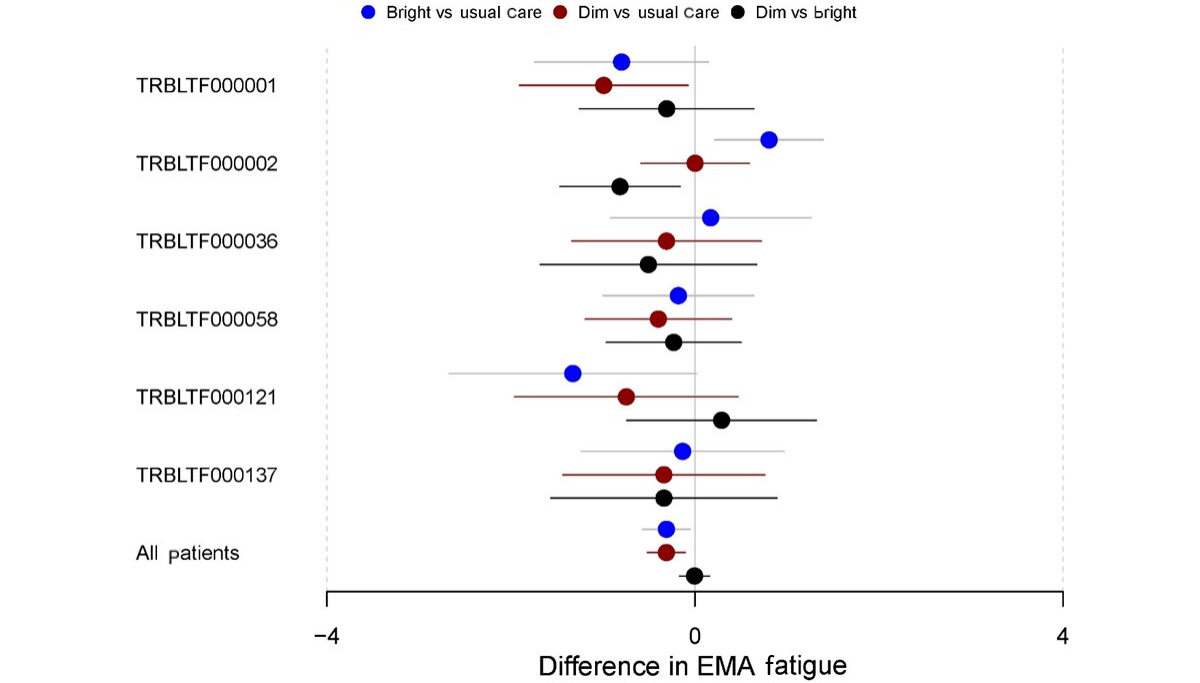
Difference in the ecological momentary assessment (EMA)–measured fatigue by intervention period (subsample of participants). Participant IDs are denoted with their deidentified study code (eg, TRBLTF000001).

To identify heterogeneity in the treatment effect between BL, DL, and UC periods in individual participants in the study, autoregressive models were used for each participant. The results for the autoregressive models on the PROMIS daily fatigue, PROMIS weekly fatigue, and EMA fatigue measures for each participant in the study can be found in Figures S1-S3 in Multimedia Appendix 2, respectively. For ease of interpretation, 6 (10%) of the 60 participants who provided data were selected to illustrate the variation in the effectiveness of the BL, DL, and UC interventions. Figure 5 displays a forest plot showing the comparative effects of each intervention period on the PROMIS daily fatigue measure for these 6 participants as well as the estimate of the effects for the full sample. This figure also shows small but significant reductions in PROMIS daily fatigue during BL versus UC periods and DL versus UC periods but no difference in BL versus DL periods for the full sample of all participants. For individual participants, however, greater reductions in daily fatigue may have been shown in BL periods than in DL periods (eg, participant TRBLTF000036), whereas others may have shown greater daily fatigue reductions in DL periods than in BL periods (eg, participant TRBLTF000002). For other participants, both the BL and DL periods showed significant reductions in daily fatigue relative to UC (eg, participant TRBLTF0000121). Many other participants showed no significant difference between any of the intervention periods. This pattern of heterogeneity was replicated across the PROMIS weekly fatigue and EMA fatigue measures (Figures 6 and 7, respectively).

**Table 3:** Participant-reported fatigue by intervention period.

Outcome	Values, mean (SD)					Values, mean difference (95% CI)		
	Baseline	UC^a^	DL^b^	BL^c^	UC vs baseline		DL vs baseline	BL vs baseline
PROMIS^d^ daily fatigue	58 (8)	54 (8)	53 (9)	53 (9)	−3.7^e^ (−5.1 to −2.4)		−5.2^e^ (−7.1 to −3.3)	−5.3^e^ (−7.0 to −3.5)
PROMIS weekly fatigue	61 (6)	58 (6)	55 (7)	56 (7)	−3.0^e^ (−4.2 to −1.9)		−5.3^e^ (−6.9 to −3.8)	−5.4^e^ (−7.1 to −3.7)
EMA^f^ fatigue	5.20 (1.74)	4.62 (1.82)	4.26 (1.91)	4.18 (1.83)	−0.62^e^ (−0.94 to −0.31)		−1.00^e^ (−1.40 to −0.59)	−1.00^e^ (−1.30 to −0.59)

^a^UC: usual care.

^b^DL: dim light.

^c^BL: bright light.

^d^PROMIS: Patient-Reported Outcomes Measurement Information System.

^e^*P* values for paired samples *t* tests: *P*<.001.

^f^EMA: ecological momentary assessment.

**Table 4:** Participant-reported fatigue by intervention period regression results.

Outcome	Values, B (95% CI)		
	Bright light vs usual care	Dim light vs usual care	Dim light vs bright light
PROMIS^a^ daily fatigue	−1.63 (−2.63 to −0.63)	−1.44 (−2.50 to −0.38)	0.20 (−0.63 to 1.03)
PROMIS weekly fatigue	−2.48 (−3.78 to −1.19)	−2.25 (−3.32 to −1.18)	0.15 (−0.84 to 1.14)
EMA^b^ fatigue	−0.31 (−0.57 to −0.05)	−0.31 (−0.52 to −0.11)	−0.01 (−0.17 to 0.16)

^a^PROMIS: Patient-Reported Outcomes Measurement Information System.

^b^EMA: ecological momentary assessment.

### Participant Satisfaction and Attitudes About Elements of the Personalized Trial

**Figure 8:**
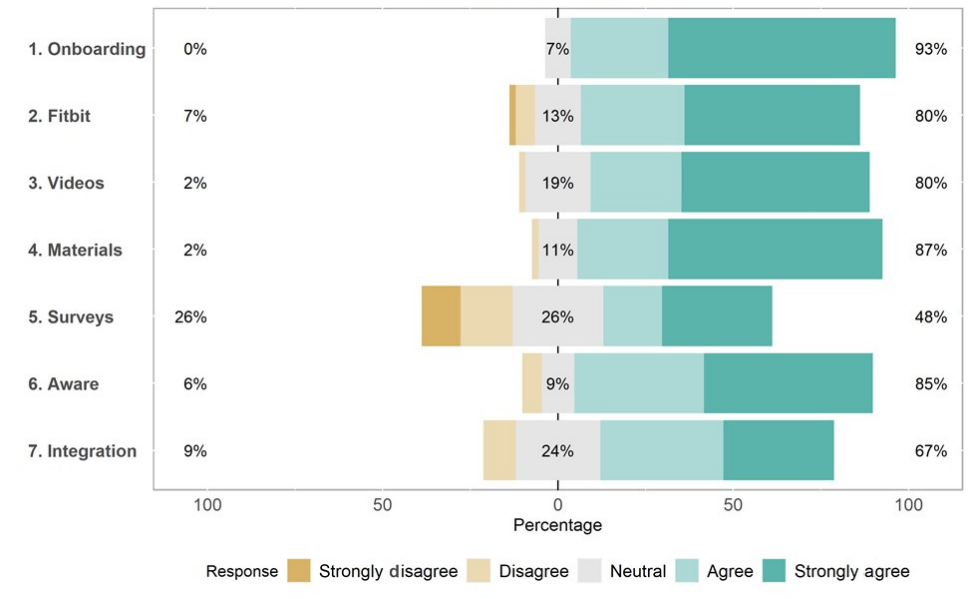
Participant satisfaction with elements of the personalized trial.

**Figure 9:**
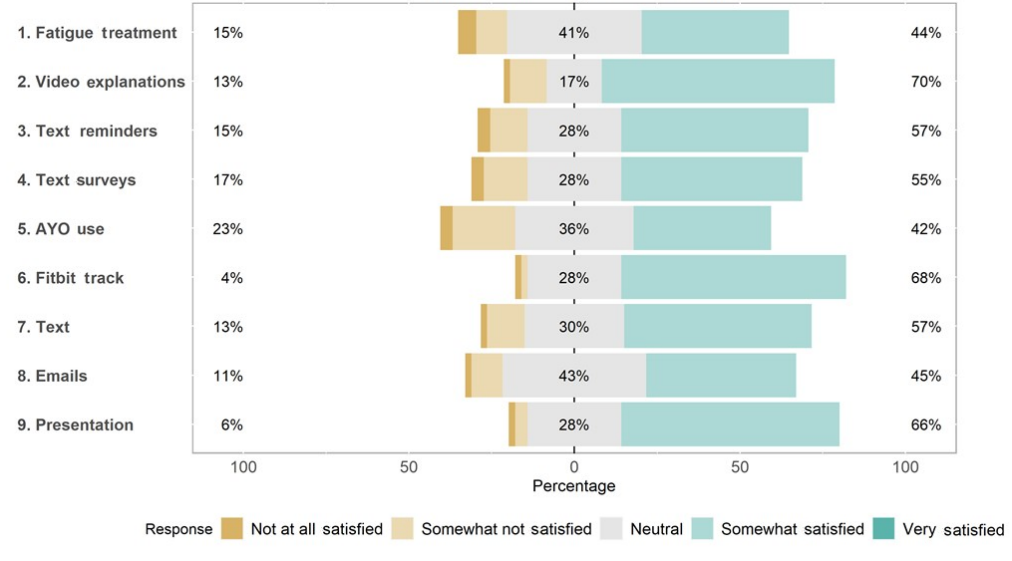
Participant satisfaction with components of the trial.

Participants who completed the satisfaction survey (53/60, 88%) demonstrated high levels of satisfaction with the elements of the personalized trial, including the consenting process, Fitbit device, study materials, and text message interventions, with average responses ranging from 3.2 to 4.6, indicating satisfaction (Table 5). Participants were most positive about the onboarding process, with 92% (49/53) of the participants rating it favorably and 8% (4/53) rating it neutrally (Figure 8). In addition, 89% (47/53) of the participants agreed that the materials they received by mail were easy to use (Figure 8). Only 42% (22/53) of the participants were satisfied with their AYO light therapy glasses (Figure 9).

Of the 53 participants completing the trial, 24 (45%) stated they would recommend the trial “strongly” and 26 (49%) stated that they would recommend the trial “a little bit.” Only 8% (4/53) of the participants stated that they would not recommend the trial to other individuals with symptoms of fatigue (Table 6). When asked how helpful participation in the trial was for symptoms of fatigue, of the 53 individuals, 17 (32%) stated that participating in the study was somewhat helpful, whereas 9 (17%) said that it was very helpful. In total, 19% (10/53) of the participants stated that it was extremely helpful (Table 6).

**Table 5:** Descriptive statistics for satisfaction measures (n=53).

Measure			Values, n (%)		Values, mean (SD; range)
**Elements of the personalized trial ^a^**					
	1.“I found the onboarding process (from the initial survey to getting my materials) for my personalized trial straightforward and easy to follow.”	53 (100)		4.57 (0.63; 3-5)	
	2.“I think my Fitbit Charge 3 was easy to use.”	53 (100)		4.20 (1.00; 1-5)	
	3.“The informational videos helped me understand how to participate in this study.”	53 (100)		4.31 (0.84; 2-5)	
	4.“The materials I received in the mail were clear and easy to use.”	53 (100)		4.46 (0.77; 2-5)	
	5.“I enjoyed receiving daily text message prompts and surveys on my cell phone.”	53 (100)		3.43 (1.37; 1-5)	
	6.“I felt like I knew what was coming next in my personalized trial.”	53 (100)		4.28 (0.86; 2-5)	
	7.“My personalized trial was easy to integrate into my daily routine.”	53 (100)		3.89 (0.96; 2-5)	
**Satisfaction with components of the trial ^b^**					
	1.“Your personalized trial of light therapy for fatigue.”	53 (100)		3.24 (0.85; 1-4)	
	2.“Video explanations and demonstrations of study devices and procedures.”	53 (100)		3.56 (0.77; 1-4)	
	3.“Text messaging for reminders.”	52 (98)		3.38 (0.84; 1-4)	
	4.“Test messaging for survey questions.”	52 (98)		3.34 (0.85; 1-4)	
	5.“Use of the AYO light therapy glasses.”	52 (98)		3.15 (0.86; 1-4)	
	6.“Use of the Fitbit Charge 3 to track your activity and sleep.”	52 (98)		3.62 (0.63; 1-4)	
	7.“Use of the study’s communication over text message.”	52 (98)		3.42 (0.77; 1-4)	
	8.“Use of the study’s communication over emails.”	52 (98)		3.32 (0.73; 1-4)	
	9.“Presentation of your results.”	52 (98)		3.58 (0.66; 1-4)	

^a^Questions rated on a 5-point Likert scale from 1 “Strongly disagree” to 5 “Strongly agree.”

^b^Questions rated on a 5-point Likert scale from 1 “Not at all satisfied” to 5 “Very satisfied.”

**Table 6:** Participant ratings of the helpfulness of the trial (n=53).

Measure		Values, n (%)
**How much would you recommend this personalized trial of light therapy to other persons with symptoms of fatigue?**		
	I would not recommend	4 (8)
	I would recommend a little bit	25 (47)
	I would strongly recommend	24 (45)
**Overall, how helpful was your participation in this study with respect to your symptoms of fatigue?**		
	Not at all helpful	6 (12)
	A little bit helpful	10 (19)
	Somewhat helpful	17 (33)
	Very much helpful	9 (17)
	Extremely helpful	10 (19)

## Discussion

### Principal Findings

The results of this trial show positive evaluations of virtually delivered personalized light therapy interventions for symptoms of fatigue. The SUS scores obtained for this trial indicated good levels of usability that were greater than or comparable with other virtual interventions targeting unsafe medication use, diabetes [46-48], obesity [49], stroke rehabilitation [50-52], spinal cord injury [53], rehabilitation of survivors of respiratory failure [54], and light therapy for sleep disturbance in Parkinson disease [55]. Ratings of the SUS in this trial were lower than those of a virtual intervention to reduce sedentary behavior after cancer surgery [56], an app-based intervention for depression [57], and light therapy for psoriasis [58]. Thus, this trial had comparable levels of usability relative to other virtually delivered interventions in the literature, including a light therapy intervention for sleep disruption. Analysis of the individual items of the SUS indicated that participants stated that they believed the trial program was easy to use, consistent, well integrated, and not overly complex. This supports the notion that this series of virtually delivered personalized trials of light therapy interventions for fatigue are highly usable and learnable. Given the importance of usability for virtual personalized N-of-1 trials, these results suggest that our system is feasible and may benefit from wider-scale implementation and use.

Across all participants, both BL and DL were associated with small but statistically significant reductions in self-reported symptoms of fatigue in both PROMIS and EMA measures. These findings were replicated across all measures of fatigue used in this study, including the daily, weekly, and EMA assessments. As in previous literature [18], participant responses to BL and DL interventions were heterogeneous. Some participants reported that both BL and DL were associated with reduced symptoms of daily fatigue (4/56, 7%), weekly fatigue (10/50, 20%), and EMA fatigue (10/55, 18%) relative to UC. Most participants reported either intervention (8/56, 14%) or neither intervention (33/56, 59%) was effective in reducing the symptoms of fatigue relative to UC. Some participants (7/56, 13%) reported increased daily symptoms of fatigue during the BL or DL intervention periods. These results suggest that BL and DL interventions are not uniformly effective across all participants and support the findings in the literature regarding the heterogeneous effects of light therapy [18].

Finally, this series of personalized interventions was rated as acceptable by the participants. The majority of the participants reported that the study and the included interventions were helpful (46/52, 88%) and would recommend participation to others if asked (49/53, 92%). Satisfaction levels with most components of the trial were also high, with the exception of the survey measures and the AYO light therapy glasses. In follow-up interviews with 47 participants, some participants expressed that they had problems with the frequency (12/47, 26%), timing (8/47 17%), and length (9/47, 19%) of the survey measures. This suggests that shorter, less-frequent surveys administered at times preferred by the participants may be more acceptable in future studies. The light therapy glasses used in this trial were not rated with high levels of satisfaction in this protocol, with 10 participants stating that the glasses were “difficult to learn to use” and another 10 stating that the glasses were “difficult to wear.” Conversely, 21 participants reported that the glasses were “easy to use.” Therefore, the participants’ acceptability ratings for the light therapy intervention were also seemingly heterogeneous. This further supports the idea that interventions for symptoms of fatigue are not “one size fits all.” Instead, personalized designs should be used to find the “best-fitting” intervention for each individual.

The fact that both the BL and DL interventions were found to have small but significant positive effects across all participants is interesting. The DL intervention had only 1% of the illuminance of the BL therapy, which might suggest that it would not be as effective. However, the findings of this study show that some participants reported greater reductions in fatigue symptoms during DL treatment periods than during the BL and UC periods. Data from the meta-analysis showed that light therapy interventions were less effective at lower levels of light intensity (measured in lux), suggesting that brighter lights would lead to greater reductions in symptoms of fatigue. However, this meta-analysis noted that color temperature (eg, blue) and light wavelength also influence the effectiveness of light therapy interventions [18]. The results of this trial show that some participants benefited more from DL therapy than from BL therapy, providing further support for the idea that the fit of an intervention with a particular participant may sometimes matter more than the theoretical strength of the intervention.

### Strengths and Limitations

This study had several notable strengths. First, the use of wearable technologies for intervention delivery (namely, the AYO glasses) and virtual assessment measures means that this type of study design is easily replicated, with N-of-1 trials being simple to scale quickly to recruit larger samples. Second, feasibility and participant satisfaction with the trial were assessed using multiple metrics, including the SUS, a validated measure of usability used to evaluate other virtual interventions. This measure was supplemented with a satisfaction survey designed to assess participants’ attitudes about the specific components of this trial. The use of the SUS and a tailored satisfaction survey allows for comparison of this trial with other similar studies, while also evaluating aspects unique to this trial.

Limitations include the type of light therapy delivered. This trial only compared 2 intensities of light (bright and dim) delivered using wearable AYO light therapy glasses. Additional data could have been obtained by varying other elements of the light therapy (including light wavelength and color temperature) as well as the light source (including stationary light sources and other wearable light sources). Altering these aspects of light therapy might provide additional information about the components of light therapy that are most effective in reducing symptoms of fatigue among individual participants.

### Conclusions

The findings of this study illustrate that a series of personalized interventions for virtual delivery of light therapy to treat symptoms of fatigue is feasible (based on usability scores on the SUS), acceptable (based on the high levels of satisfaction), and effective (based on reduction in symptoms of fatigue). Ratings of feasibility and acceptability varied by specific elements of the personalized trials and thereby provided information to refine and improve the intervention. Both BL and DL therapies were found to have small but significant effects on the reduction of fatigue symptoms; however, the effect sizes for BL and DL therapies varied greatly across participants. This supports the need for personalized trials to identify which light therapy interventions are most effective for a given individual and to guide treatment selection. Future studies are needed to evaluate the effectiveness of interventions selected to reduce the symptoms of fatigue using personalized trials. If personalized trials can help an individual select the treatment that works best for them to reduce their symptoms of fatigue, there is potential for broader application of personalized methods in fatigue treatment.

## Appendix

Multimedia Appendix 1 Consent form.

Multimedia Appendix 2 Autoregressive models on the Patient-Reported Outcomes Measurement Information System (PROMIS) daily fatigue, PROMIS weekly fatigue, and ecological momentary assessment (EMA) fatigue measures.

## Abbreviations

AR (1): autoregressive model of order 1
BL: bright light
CENT: CONSORT extension for reporting N-of-1 Trials
CONSORT: Consolidated Standards of Reporting Trials
DL: dim light
EMA: ecological momentary assessment
PROMIS: Patient-Reported Outcomes Measurement Information System
SUS: System Usability Scale
UC: usual care

## Data Availability

The trial protocol, deidentified data, and analysis code for this trial are publicly available on the Open Science Framework [59]. Requests for additional data may be made to the corresponding author. Data requests will be reviewed by the Northwell Health regulatory team, and access to full data will be granted following institutional review board approval, as applicable, and the completion of a data use and sharing agreement with Northwell Health.
