# Supplementary material for: A Series of Personalized Virtual Light Therapy Interventions for Fatigue: Feasibility Randomized Crossover Trial for N-of-1 Treatment
Source: JMIR Form Res. 2023 Sep 18;7:e45510. doi: 10.2196/45510 (PMC10546268; doi:10.2196/45510)
Supplement: Multimedia Appendix 1 [file formative_v7i1e45510_app1.pdf]

APPENDIX 1 – Sample Consent Form

Northwell Health

Consent for Participation in a Research Study

Study Title: Re-engineering Precision Therapeutics Through N-of-1 Trials: Feasibility Study of Personalized Trials of Light Therapy for Fatigue

Principal Investigator: Karina W. Davidson, PhD, MASc

Sponsor: National Institutes of Health (NIH)

About this research
You are being asked to participate in a research study pilot. A pilot study is a small-scale study that explores the reasonableness of doing the type of research.

This consent form will give you information about the study to help you decide whether you want to participate. Please read this form, and ask any questions you have, before agreeing to be in the study.

Important Information
This information gives you an overview of the research. More information about these topics may be found in the pages that follow.

**Why am I being asked to provide my consent?**	This is a pilot research study, which is different than personal medical care. Scientists do research to answer important questions which might help change or improve the way we do things in the future.
**Do I have to join this research study?**	No. Taking part in this pilot research study is voluntary. You may choose not to take part in the pilot study or may choose to leave the pilot study at any time. Deciding not to participate, or deciding to leave the pilot study later, will not result in any penalty or loss of benefits to which you are entitled.<br><br>This study may enroll employees of Northwell Health. Employee participation or non-participation will have no bearing on an employee's position at Northwell Health.<br><br>This study may also enroll students, including those that may attend Hofstra University. Student participation or non-participation will have no bearing on a student's grades nor academic standing.

**Why is this research study being done?**	The main purpose of this pilot study is to test the feasibility and satisfaction of methods used for Personalized Trials (meaning your own personal response or N-of-1), including:<br>• Methods used to remotely recruit and enroll participants,<br>• Using online web and social media advertising to recruit and enroll participants in research study<br>• Methods used to collect and assess symptoms in response to a health and wellness strategy (in this case, blue light).<br><br>Our main goal is to see if an N-of-1 study design, or what we are terming Personalized Trials, can have widespread use in future research and clinical practice. We are not testing the safety or the effectiveness of the light therapy devices.
**What will happen to me during the study?**	In this pilot study, you will test the effects of two types of light therapy devices for chronic fatigue, a bright light and a dim light. Some weeks, you will use the bright light glasses for 30 minutes each morning, and some weeks you will wear the dim light glasses for 30 minutes each morning. Other weeks you will be instructed not to participate in any light therapy, and instead asked to treat your fatigue as you normally would. Each day you will answer questions about the fatigue you are experiencing, and some questions about your fatigue, sleep, stress, pain, concentration, confidence, and mood. You will also wear a Fitbit® each day and night to track your activity and sleep. You will receive a participant report that summarizes your observed response while you used both light therapy interventions. At the end of the study, we will send you a satisfaction survey and ask you to participate in a phone interview to share your opinions about your Personalized Trials experience.
**How long will I participate?**	The Personalized Trial will take place over the course of 14 weeks. You will be asked to complete a satisfaction survey after you receive your summary results (within 3 months of the Personalized Trial being complete). You will also be asked to participate in a 1-hour phone interview to talk about your opinions about your experience. Study participation will end upon completion of the phone interview.
**Will taking part expose me to risks?**	Some people may experience mild side effects from light therapy. Possible side effects may include headache, dry-mouth, eyestrain, nausea, or hyperactivity. Hyperactivity means being unusually active. These side effects normally go away quickly on their own. If you experience severe side effects, you should immediately stop light therapy and contact a member of the study team.<br><br>There may be a chance that blue light may worsen glaucoma, a condition of increased pressure in the eyeball causing gradual loss of sight.

	Although there is no evidence of this happening to humans, some scientists have seen a worsening of glaucoma in controlled animal studies with mice. Even though we believe the blue light used in this study does not carry the same risk as the blue light used the mice studies, individuals with glaucoma are not eligible to participate.<br><br>Another risk of taking part in this pilot study is the possibility of a loss of confidentiality or privacy. Loss of privacy means having your personal information shared with someone who is not on the study team and was not supposed to see or know about your information. The study team plans to protect your privacy. Their plans for keeping your information private are described in the sections below.
**Are there any benefits to participation?**	A Personalized Trial of Light Therapy for Fatigue is not designed to benefit you directly. You may or may not experience anything when wearing either pair of glasses but in receiving your observed data trends when using either pair of glasses, you will find out information about your own personal response to light therapy which may give you information on how to manage your fatigue We cannot promise any benefits to others from your taking part in this research. However, possible benefits to others include demonstrating that a Personalized Trial design can help doctors and scientists discover new ways to help patients in the future.
**What are my alternatives to participation?**	You do not need to participate in this pilot study to try light therapy. There are many light therapy devices available for purchase online, including the AYO light therapy glasses used in this study.

Please review the rest of this document for details about these topics and additional things you should know before making a decision about whether to participate in this research.

Introduction

You are being asked to join a pilot research study. The purpose of a pilot research study is to answer specific questions.

You do not have to be in this pilot study to receive medical care. You should ask questions before you decide if you want to participate. You can also ask questions at any time during the pilot study by emailing **______________** or calling __________.

Why is this research study being done?

The main purpose of this pilot study is to assess the feasibility and satisfaction of methods used for Personalized Trials, including:

- Methods used to remotely recruit and enroll participants,
- Using online web and social media advertising to recruit and enroll participants in research study

- Methods used to collect and assess symptoms in response to a health and wellness strategy (in this case, light therapy).

This data will be used to inform future Personalized Trials, and it will help us understand the needs for a future technology platform to conduct these trials. We are testing these methods and technology to help determine if a Personalized Trial design, or a research study that occurs in only one patient, can have widespread use in future research and clinical sessions.

This particular pilot study will test a Personalized Trial design to see if it can be useful in observing trends in light therapy for symptoms of fatigue. We are not testing the safety or the effectiveness of the light therapy devices. You are being asked to participate in this pilot study because you have self-identified as someone who often has symptoms of fatigue.

You will not be able to participate in this study if:

- You are 60 years old or older or younger than 18 years of age
- You are pregnant
- You have had a diagnosis of a serious mental health condition or psychiatric disorder, such as bipolar disorder
- You have had a previous diagnosis of eye disease, such as cataracts, glaucoma, macular degeneration, Stargardt or family history of Stargardt, retinitis or retinopathy, or other retinal disorders
- You have had a previous diagnosis of diabetes
- You have sensitivity to light or use of medication causing sensitivity to light
- You have epilepsy or a history of seizures
- You participate in shift work (evening/night shifts, early morning shifts, rotating shifts, etc.)

You live outside of the United States

How many people will take part in this study?

This pilot research study hopes to enroll up to 150 people. Up to 60 participants will be randomized (like the flip of a coin) to complete research procedures.

How long will I be in this study?

If you are selected to take part in this pilot study, the Personalized Trial procedures will last for 14 weeks. When all of your data is collected, you will be sent a custom participant report along with a satisfaction survey. Once you complete the satisfaction survey, you will be asked to take part in a follow-up phone interview to discuss your experience with a member of the study team.

If you will be traveling outside of the United States during the pilot study period, or if you will not have access to text-messaging or internet for more than a few days during the pilot study period, you should talk to a member of the study team to determine your eligibility for this study.

What will happen in this research study?

After filling out this consent form, you will be asked to provide the research team some more information about yourself. This will include your cell phone number, cell phone carrier, and home address. This pilot study uses text-message reminders to help you through the study protocol. Completing the surveys will require cellular data if you are not connected to Wi-Fi. You will not be reimbursed for text messages or data charges, and standard carrier rates may apply.

If you are selected as a potential research participant in this pilot research study, you will be mailed your first study kit. This will include a Fitbit Charge 3™ and printed materials to help guide you through this pilot study. You will be asked to download an app to your personal phone in order to use the Fitbit Charge 3™. You will be sent an email to confirm your pilot study start date, and you will also receive a text message the day that your study begins. You will be asked to download the Fitbit® app to your phone to collect your data. Any e-mail correspondence that you send to the study team or the study team sends to you during your participation in the study, other than the custom report of your trial experience, will be unencrypted. This means that others may be able to access the information and read it once it is transmitted over the internet. Any e-mail correspondence sent by the study team will reference "the fatigue study" or "the personalized trial of blue light for fatigue." The fact that you have fatigue and are participating in the study constitutes protected health information. Other than the reference to the study title, e-mail correspondence from the study team will not contain protected health information.

The first two weeks of the study is considered your baseline period. The purpose of this baseline period is to help us determine your usual activity and how you normally feel, without using any blue light. You will not receive any light therapy during your baseline period. Each evening you will receive a survey asking you a few questions about your fatigue that day, and your previous night's sleep. This survey will take approximately 5 minutes to complete. At three random times each day, you will receive a text message asking you to rate your pain, fatigue, stress, mood, confidence, and concentration levels at that exact moment. This survey will take approximately three minutes of your time. On Sunday evenings, you will receive a slightly longer survey asking you to reflect on your fatigue that week. This survey will take less than 10 minutes to complete.

You will be asked to wear your Fitbit Charge 3™ all day and night, even while you are sleeping. Your Fitbit® can be charged during periods of extended sitting, like when you are in your car or sitting at a desk, or while you are showering. Every two days you will need to sync your Fitbit® device. You can sync your device by opening the Fitbit® app and waiting for your data to load.

It is very important that you wear your Fitbit® device all day and night, that you sync your Fitbit® device at least every two days, and that you answer the survey questions sent to you each day. You will be able to select the best times to send you these messages before your baseline period begins, and you will have the opportunity to change these times if there is a change in your schedule. You may be asked to discontinue your participation in this study if you are not wearing your Fitbit® long enough, or if you do not answer enough survey questions each day.

This is often referred to as adherence or compliance, and will be measured by at least 80% satisfactory completion.

If there is enough data collected during your baseline period, you will be contacted by a member of our pilot study team by email with your treatment schedule for the rest of the study. You will be mailed a second study kit, which will include two AYO light therapy glasses. The glasses will be labeled “bright light” or “dim light” and will be used during your light therapy treatment weeks. Both bright and dim light glasses have four (4) light emitting diodes (LEDs) that put out light within the visible spectrum of blue light at 470 nanometers (nm), with different levels of energy output (irradiance). You will be asked to download a study app to your personal phone in order to use the AYO light therapy glasses. You will be assigned a random order of 6 treatment periods. Each treatment period will last 2 weeks. A treatment period may consist of bright blue light therapy, dim blue light therapy, or usual care. Usual care is a treatment period where you will be asked to not use either pair of light therapy glasses, but still answer questions and wear your Fitbit®. You will receive a calendar and text message reminders that tell you when you are assigned to each intervention or usual care. We will also be able to monitor your use of the light therapy devices. If we see that you are not using the glasses appropriately, we will contact you by text message to remind you of your assigned treatment schedule and duration of use.

During treatment weeks, you will be also be asked to continue wearing your Fitbit® device each day and night. You will continue to receive 4 text messages every day with survey questions about your fatigue, sleep, pain, stress, mood, concentration, and confidence levels. You will also be asked about any treatment-related side effects you may be experiencing. If you are concerned about any side effects, you may stop doing light therapy at any time and contact a member of the research team for more information about continuing your study participation. You will also continue to receive a slightly longer weekly survey at the end of each week reflecting on your fatigue from the last week.

During the pilot study period, you may also receive additional text messages to remind you of next steps for this study, or to ask you to sync your data if it is not appearing correctly. We will send a maximum of 6 text messages per day during the study, including these reminders and the survey questions outlined above.

The treatment portion of the study will end once you have completed one baseline period (two weeks), two periods of bright light therapy (two weeks each), two periods of dim light therapy (two weeks each), and two periods of usual care (two weeks each), or 14 weeks total. Your participation in this pilot research study is voluntary. You may stop participating at any time by the methods described in the relevant section below. Alternatively, you may be asked to end your study participation by a member of our research team for any reason.

We will ask you to return 1 of the AYO light therapy glasses at the end of the study. The light therapy glasses can be returned in the padded envelope you received in your second personalized

trials box using the return slip that was also provided to you. No personal information, such as your name or address, will be printed on these materials.

We will compile the data from your questionnaires, the AYO light therapy glasses, and your Fitbit®. Any identifying information about you will be removed. A statistician will then analyze your coded data. Only the research team will have the key to identify you based on your research code. We will then turn this analysis into a report of your observed data and symptoms over the study period. Creating this type of report helps us to see if a Personalized Trial design works or is feasible. You will be sent this report in an encrypted email by our study team. This report is not meant to offer medical advice, but you may find that the information is useful for your own knowledge because it could help you understand your personal response to each light therapy. You will be sent a satisfaction survey within one week of receiving your report.

Within one month from your study ending, regardless of when that takes place, you will receive a phone call from a member of our study team to talk about your experience as a research participant. We will ask you several questions about what it was like to participate in the study. We will also ask about your opinion on Personalized Trials.

Your trial is complete after you completed 14 weeks of data collection and you have completed your satisfaction survey and phone interview. Upon full completion of the trial, including submission of your satisfaction survey and phone interview, you will receive a $100 pay card (ClinCard) as a thank you for your participation. You can use this card like a credit or debit card anywhere MasterCard is accepted, including online.

What are the risks of the research study? What could go wrong?
Although this is a minimal risk pilot study, there are some potential risks to participating.

<u>Treatment Side Effects</u>
Blue light therapy was selected for this study because it has been shown to be safe and commonly used by consumers to address fatigue with minimal side effects. There may be a chance that blue light may worsen glaucoma, a condition of increased pressure in the eyeball causing gradual loss of sight. Although there is no evidence of this happening to humans, some scientists have seen a worsening of glaucoma in controlled animal studies with mice. Even though we believe the blue light used in this study does not carry the same risk as the blue light used the mice studies, individuals with glaucoma are not eligible to participate.

There may be risks to prolonged exposure of blue light that are unknown at this time. You should use the blue light glasses as directed to lessen the chance of any unknown risks.

Common side effects some people may experience are headache, dry mouth, eye-strain, nausea, or hyperactivity. Hyperactivity means being unusually active. These side effects are normally very mild and brief.

If you are concerned about any of these side effects or any others you may experience, you should immediately stop doing light therapy and contact a member of the study team. You may still be able to continue with the study with modified light therapy treatments, or you may be asked to end your participation in the study.

<u>Loss of Confidentiality or Privacy</u>

One risk of taking part in this pilot study is the possibility of a loss of confidentiality or privacy. Loss of privacy means having your personal information shared with someone who is not on the study team and was not supposed to see or know about your information. The study team plans to protect your privacy by only sharing necessary information about you to those outlined in the "Who else will see your information?" section below.

Any information collected during this study that can identify you by name will be kept confidential. We will separate your personal information (name, address, cell phone number) from all the information you provide us (this is called giving a "code" to your data). The key to your identifiable information will be stored separately in a secure, password-protected, HIPAA compliant database. Your personal or identifiable information is not stored on any of the study devices used in this study.

Your questionnaire responses will be obtained by a text message link from a secure web application that is used to collect survey data. We will do everything we can to keep your data secure, however, complete confidentiality cannot be promised.

What are the benefits of this research study?

This Personalized Trial of Light for Fatigue is not designed to benefit you directly. You may or may not experience anything when wearing either pair of glasses but in receiving your observed data trends when using either pair of glasses, you will find out information about your own personal response to light therapy which may give you information on how to manage your fatigue. We cannot promise any benefits to others from your taking part in this research. However, possible benefits to others include demonstrating that a Personalized Trial design can help doctors and scientists discover new ways to help patients in the future.

Will I receive my results?

Although the main purpose of this pilot study is to assess the methods for future Personalized Trials, the data we collect from you during study activities could impact how you treat your symptoms of fatigue. For this reason, much of the information collected about you during the study will be analyzed and provided back to you. You will receive this data in the form of a participation report. The data included in the participation report will include graphs and statistics about your self-reported fatigue, self-reported stress, self-reported pain, mood, confidence, and concentration, activity patterns, sleep patterns, and adherence (or how closely you followed the study procedures). This data will be shown in relation to weeks you had bright blue light therapy, the weeks you had dim blue light therapy, and the weeks you had usual care. This report is meant to summarize your observed data trends and self-reported symptoms, and should not be considered medical advice.

If you do not want to take part in this research study, what are your other choices?
Since this project is assessing the Personalized Trial study design, the alternative is not to participate.

If you are interested in learning about effective treatments for your symptoms of fatigue, or if you want to learn more about your personal results, you may wish to meet with professionals with expertise to help you learn more about available treatments. The study team/study will not cover the costs of any follow-up consultations or actions.

Are there any costs for being in this research study?
This pilot research study is funded by the National Institutes for Health (NIH). All study related devices will be provided to you at no cost. This includes the Fitbit Charge 3™ and AYO light therapy glasses. Neither you nor your insurance company will be billed for your participation in this research.

This pilot study uses text messages to deliver links to notifications, reminders, and study questionnaires. Standard message and data rates from your wireless carrier may apply. You will not be compensated for any costs related to data usage or sending or receiving text messages by the study or by members of the study team.

Will you receive any payments for participating in this research study?
After successfully completing all aspects of this study, you will be mailed a $100 payment card called a ClinCard that can be used like a credit or debit card anywhere MasterCard is accepted, including online. As a thank you for participation, you will also be able to keep your Fitbit Charge 3™ (a value of $120), and one of the sets of light therapy glasses (a value of $299).

What are your rights as a research participant?
Your participation in this project is voluntary. The quality of your medical care and/or employment or enrollment status will be the same, whether you join, refuse to join, or decide to leave the pilot study.

If you do not join the study you will not be penalized or lose benefits to which you are entitled. If you join the study you may withdraw at any time without prejudice to your future care and/or employment at Northwell Health.

This study may enroll employees of Northwell Health. Participation or non-participation will have no bearing on your position at Northwell Health.

This study may also enroll students, including those that attend Hofstra University. Student participation or non-participation will have no bearing on your grades or standing at your academic institution.

Could you be taken off the study before it is over?
It is also possible that your participation in this pilot study may end without your consent. This decision may be made by a researcher, study sponsor or the Institutional Review Board, (the committee that oversees research at this institution).

Reasons for withdrawal may include:

- Failure to follow instructions, including maintaining less than 80% adherence to survey responses and Fitbit® use
- Failure to complete your light therapy treatments
- Significant cell phone carrier issues that prevent you from receiving study text messages
- It is not in your best interest to continue on this pilot study, or
- The pilot study is stopped

If you withdraw from this pilot study or if you are withdrawn from the pilot study, any data already collected will continue to be used. However, no new data will be collected.

What happens if new information is learned?
You will be told of any new findings that may change your decision to continue to participate. Your consent to continue to take part in this pilot study may be obtained again.

What information will be collected and used for this study?
If you agree to be in this pilot study, we will collect information that identifies you. We may collect the results of questionnaires, interviews, Fitbit® activity and sleep, light therapy use via AYO, and video views. We will only collect information that is needed for the research. This information has been described in this consent form. If you sign this consent form, you are giving us permission to collect, use and share your health information.

<u>Fitbit®</u>
Data collected by Fitbit® includes activity data (steps, activities, intensity, heart rate, floors climbed), sleep data (total sleep minutes, sleep stage estimates, sleep and wake times), and device data (last sync date and Fitbit® battery level). No information that can be used to identify you will be associated with your Fitbit® study account. We will only collect this data through the 14-week pilot study period. Once your study period is complete, you will be sent instructions on how to un-link your Fitbit® device from the account.

<u>AYO</u>
Data collected by the AYO light therapy glasses includes session number, date and time a device was used, the type of device that was used (in other words, bright blue or dim blue), and how long the device was used. No information that can be used to identify you will be associated with your AYO study account. We will only collect this data through the 14-week study period. Once your study period is complete, you will be sent instructions on how to continue using the AYO glasses after the study ends if you so choose.

N1Thrive
Survey data will be collected via a secure web browser and stored in a HIPAA-compliant, Northwell approved database. Text messages will alert you to a new message from "N1Thrive" and contain a link to open this secure browser directly on your phone. No identifying information will be shared via text message.

What information will be shared outside of Northwell Health?
Your privacy is important to us. We will only share information that is necessary to complete the study.

AYO
If you agree to be in this pilot study, and if you are selected as a participant, you will be sent two pairs of AYO light therapy glasses. You will also be sent instructions on how to connect the device to a study application account. General user IDs that have been created for this research study have been used to create AYO study accounts without any information that could identify you. Only the research team will have the key to connect you to your general AYO User ID. It is important that you use the AYO account provided during the study to protect your information, and to allow us to collect your data. The research team will not share any of your personal information with AYO.

Fitbit®
If you agree to be in this pilot study, and if you are selected as a participant, you will be sent a Fitbit Charge 3™. You will also be sent instructions on how to connect the device to a study account. General email addresses that have been created for this research study have been used to create these study accounts without any information that could identify you. It is important that you use the Fitbit® account provided during the study to protect your information, and to allow us to collect your data. The research team will not share any of your personal information with Fitbit®. At the end of the study, you will be asked to remove the study account from your phone. If you would like to keep the Fitbit Charge 3™, you will be sent instructions on how to create your own Fitbit® account to connect the device.

Fitabase™
Data will be shared from your Fitbit® to the research team using an online portal called Fitabase™. The study account given to you to connect your Fitbit Charge 3™ will be linked to an identification number in the Fitabase™ system. No information that could be used to identify you will ever be shared with Fitabase™. Only the research team will have access to data that will be able to connect a research participant to their Fitabase™ ID. Fitabase™ will stop sharing your data at the end of your study, but as an added step, you will be asked to remove the study account from your device if you would like to keep your Fitbit Charge 3™.

N1Thrive
N1Thrive, the company the study team is partnering with to develop the technology to analyze data for current and future Personalized Trials, will send study messages via a HIPAA-compliant manner on behalf of the study team. The study team will have direct access to the data shared

through N1Thrive. At the end of the study, your identifying information will be removed from the N1Thrive database.

Who else will see your information?
Study records that identify you will be kept private. You will not be identified in study records or publications disclosed outside Northwell Health, except as detailed below.

Investigators will share information collected from this pilot research study with:
- Study sponsor (NIH) and/or its agents,
- Other researchers,
- Accrediting agencies,
- Data safety monitoring board.

The following reviewers may access your study records to make sure that this study is being done properly:
- Representatives from federal and state government oversight agencies, such as the Department of Health and Human Services, and the National Institutes of Health
- Representatives from Northwell Health's Human Research Protection Program, (a group of people that oversee research at this institution)

In the future, we may publish results of this pilot study in scientific journals and may present it at scientific meetings. If we do, we will not identify you.

If the researchers learn about potential serious harm to you or someone else or other public health concerns, it will be shared with the appropriate authorities.

Can you change your mind?
If you change your mind about being in the pilot study, you may withdraw at any time. If you want to withdraw from the study, you need to send an email to the researcher at the following address: __________________. Alternatively, you can send a letter to the researcher at the following address:

Dr. Karina W. Davidson
Center for Personalized Health
____

Your email or letter needs to say that you have changed your mind and do not want to continue to participate. You may also need to leave the pilot research study if we cannot collect any more health information. We may still use the information we have already collected. We need to know what happens to everyone who starts a research study, not just those people who stay in it.

Will information about this study be available to the public?

The researcher also plans to share information about the pilot study, including de-identified data, on the following data sharing website: https://cos.io/. The Open Science Framework is a free, open-source web application built to provide researchers with a free platform for data and materials sharing. There will be no identifiable data (like your name) posted to this website or used in future studies.

In addition, a description of this clinical trial will be available on http://www.Clinical Trials.gov , as required by U.S. Law. This Web site will not include information that can identify you. At most, the Web site will include a summary of the results. You can search this website at any time.

Certificate of Confidentiality
To help us protect your privacy, this research is covered by a Certificate of Confidentiality from the US Department of Health and Human Services (DHHS). The Certificate of Confidentiality means that researchers cannot be forced to identify you, even under a court subpoena. The Certificate does not mean the Secretary of DHHS approves or disapproves of the project. It adds special protection for the research information about you. You should know, however, that researchers may provide information to appropriate individuals or agencies if harm to you, harm to others or child abuse becomes a concern. A Certificate of Confidentiality does not prevent you or a member of your family from voluntarily releasing information about yourself or your involvement in this research. However, if an insurer, employer or other person learns about your participation and gets your consent to receive research information, then the researchers will have to provide your information.

Will my information be used for research in the future?
Information collected from you for this research may be used for future research studies or shared with other researchers for future research. If this happens, information which could directly identify you will be removed before any information is shared. Since identifying information will be removed, there will not be an additional consent for future research. By consenting to participate in this study you are agreeing to allow your coded data to be used by future researchers without additional consent.

Some information collected during this pilot study that can identify you will be kept on file. This information may be used in the future to contact you for future participation in Personalized Trials. This information will be stored on a secure database. It will only be accessible by trained members of the study team. If you change your mind about being contacted in the future, you may follow the procedures outlined above to notify the researcher.

Does the investigator of this study receive money if you take part?
The investigators on this pilot study receive money to conduct the study, but do not financially benefit from your participation. The money they receive is to pay them back for the costs of conducting the research study. Funding for this research study is provided by the National Institutes of Health (NIH).

Who can answer your questions about this study?
If you have any questions about the pilot study, you may call Alexandra Perrin, Clinical Research Assistant, at _____________ or email ____________. If you have questions about side effects or injury caused by research you should call Joan Duer-Hefele RN, MA, CCRC, Director of Clinical Research, at _____________. If you need emergency care, dial 911 or go to the nearest Emergency Room. If you have questions about your rights as a research participant, concerns about being in the study, or would like to offer input, you may contact the Office of the Institutional Review Board at ______________.

A signed copy of this consent form will be mailed to you.

[Signature Page Follows]

Please respond to the following questions to demonstrate your understanding of study procedures and your rights as a research participant.

1. As a participant, I will need to wear my Fitbit® 24 hours a day, even while I am sleeping.

   ☐ True

   ☐ False

2. As a participant, I will use light therapy glasses every morning for 8 weeks out of the 14 week study, as instructed.

   ☐ True

   ☐ False

3. As a participant, I can remove myself from the pilot study at any time by contacting the researcher.

   ☐ True

   ☐ False

4. As a participant, I will receive at most 6 text messages a day, unless there is an unexpected problem with my data.

   ☐ True

   ☐ False

Summation/Signature

☐ I consent to be a part of the n1thrive Personalized Trial of Light Therapy for Fatigue Research Study

By checking the box and signing this form, you are consenting to the information found at personalizedhealth.org/fatigue-consent-form-11-20-2020. You have read the above description of the research study. You have been told of the risks and benefits involved and all your questions have been answered to your satisfaction. A member of the research team will answer any future questions you may have. You voluntarily agree to join this study and know that you can withdraw from the study at any time without penalty. By signing this form, you have not given up any of your legal rights.

____ ____________________

Electronic Signature Date & Time
Stamp
