# Supplementary material for: A Series of Personalized Virtual Light Therapy Interventions for Fatigue: Feasibility Randomized Crossover Trial for N-of-1 Treatment
Source: JMIR Form Res. 2023 Sep 18;7:e45510. doi: 10.2196/45510 (PMC10546268; doi:10.2196/45510)

***Figure S1.*** *Difference in PROMIS Daily Fatigue by Intervention Period (Full Sample)*

*
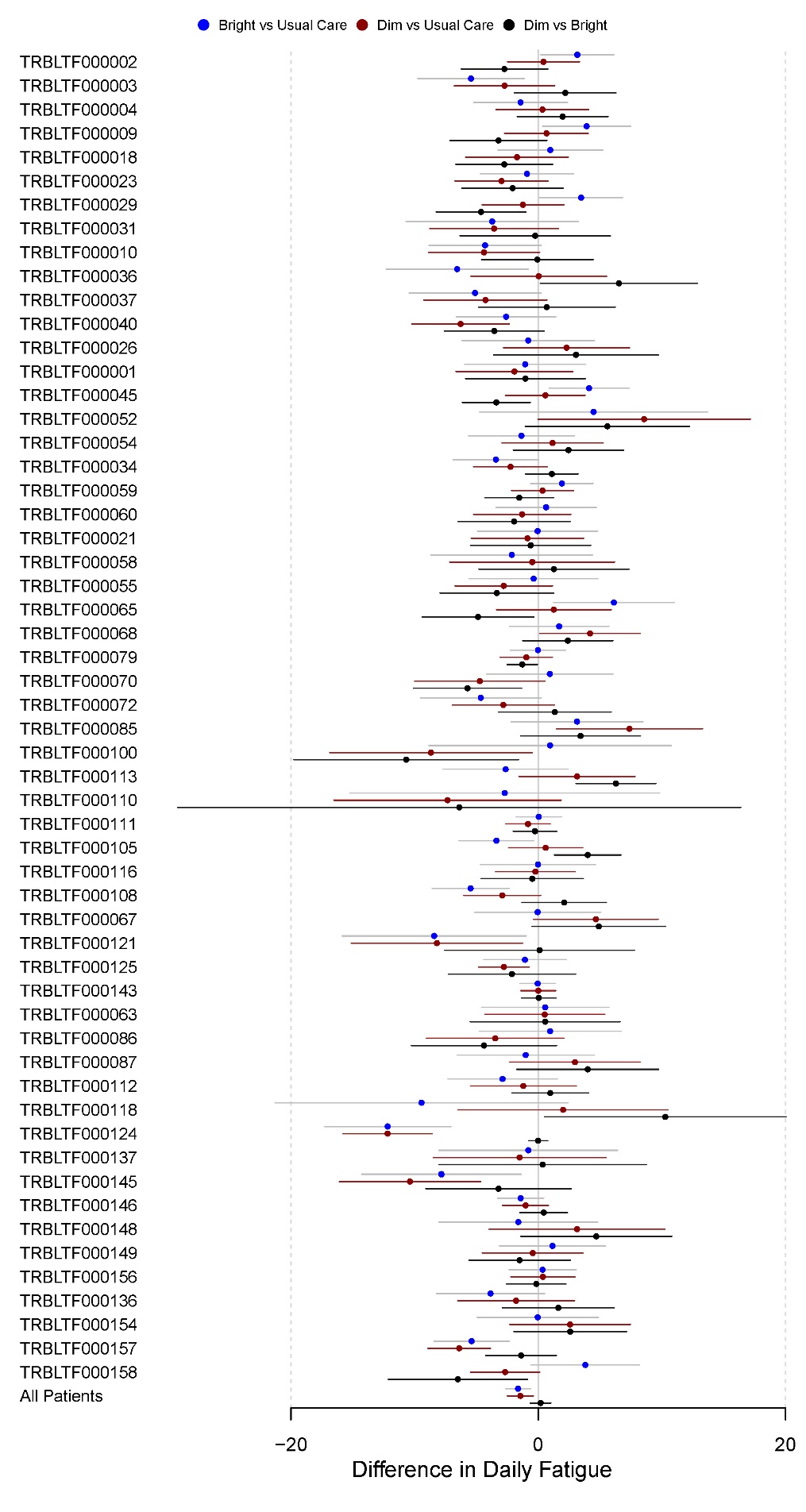
*

***Figure S2.*** *Difference in PROMIS Weekly Fatigue by Intervention Period (Full Sample)*

*
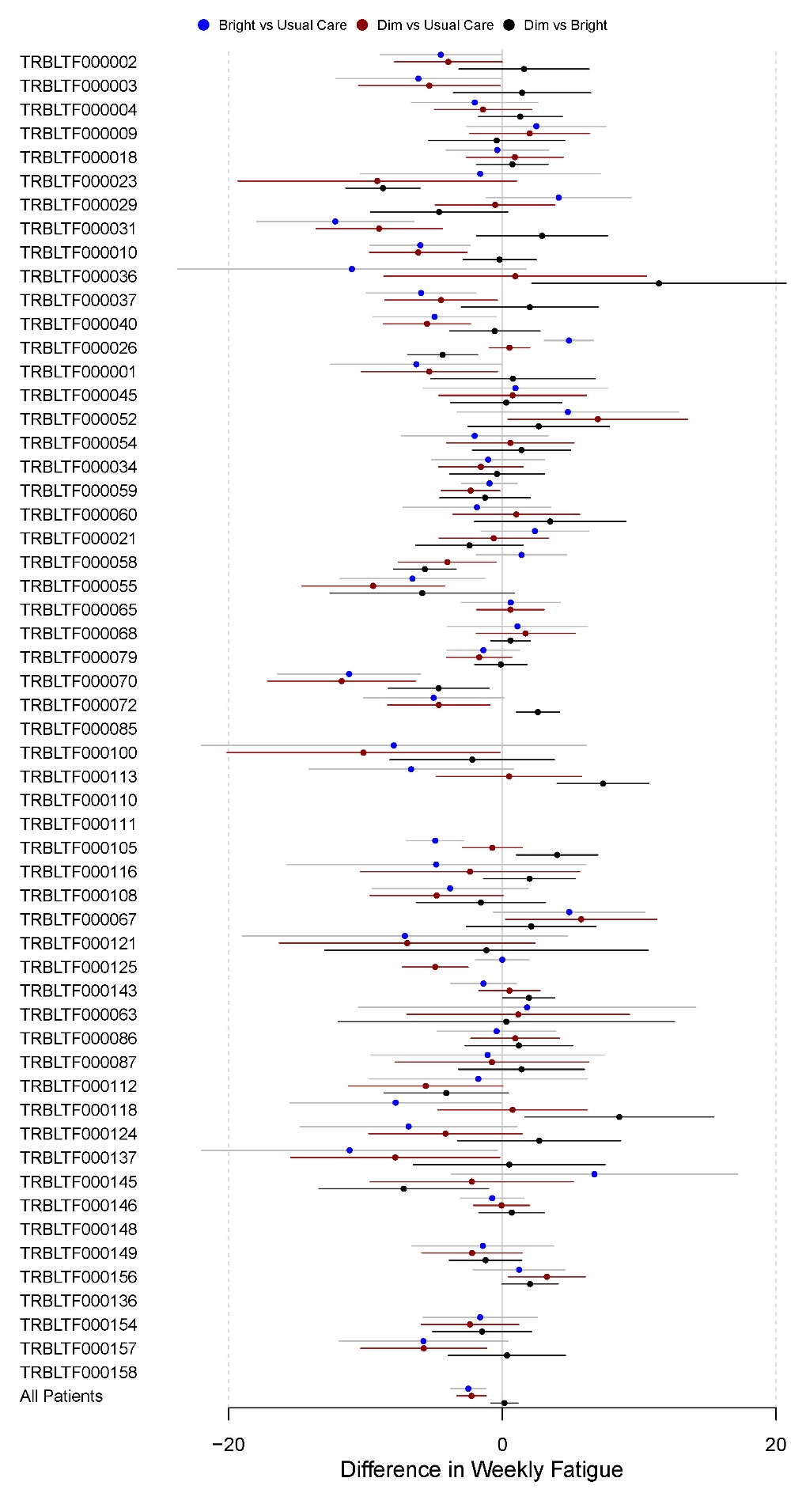
*

***Figure S3.*** *Difference in Ecological Momentary Assessment (EMA) Measured Fatigue by Intervention Period (Full Sample)*


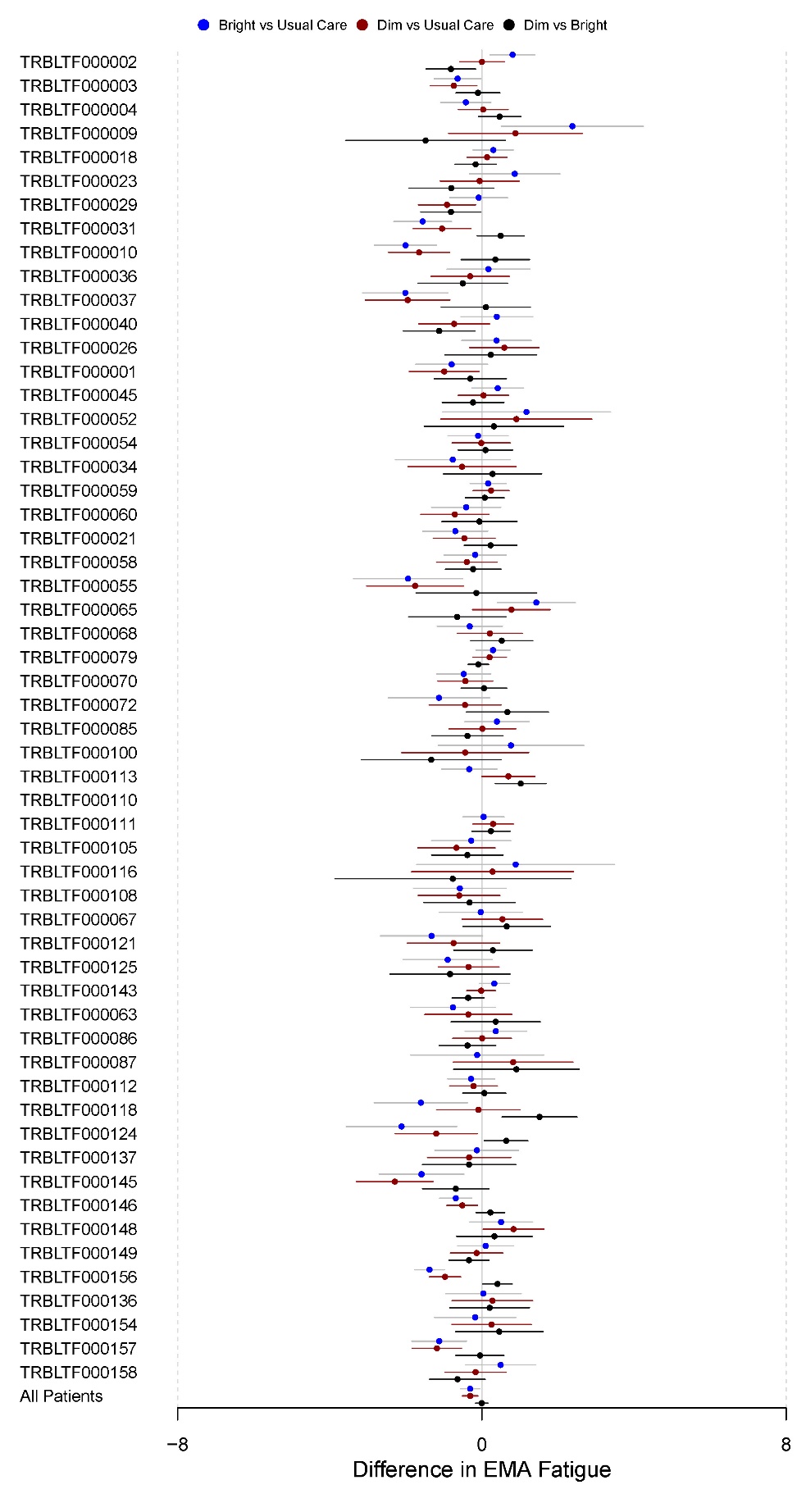

Supplement: Multimedia Appendix 2 [file formative_v7i1e45510_app2.docx]
